# Supplementary material for: The bile acid-sensitive ion channel is gated by Ca2+-dependent conformational changes in the transmembrane domain
Source: bioRxiv. 2025 Jan 11:2025.01.10.632481. Preprint. [Version 1] doi: 10.1101/2025.01.10.632481 (PMC11741473; doi:10.1101/2025.01.10.632481)
Supplement: Supplement 1 [file media-1.pdf]

Supplementary Information

The bile acid-sensitive ion channel is gated by Ca^{2+}-dependent conformational changes in the transmembrane domain

Makayla M. Freitas[1] and Eric Gouaux[1,2*]

[1]Vollum Institute, Oregon Health and Science University, 3232 SW Research Drive, Portland, OR, USA.

[2]Howard Hughes Medical Institute, Oregon Health and Science University, 3232 SW Research Drive, Portland, OR, USA.

*Correspondence to: Eric Gouaux, gouauxe@ohsu.edu

Supplementary Information

Supplementary Fig. 1: Function of hBASIC construct.

Supplementary Fig. 2: Biochemical and functional characterization of recombinant hBASIC.

Supplementary Fig. 3: Cryo-EM processing pipeline for 2 mM Ca^{2+} data set.

Supplementary Fig. 4: 2mM Ca^{2+} hBASIC cryo-EM map analysis.

Supplementary Fig. 5: Comparison of hBASIC to cASIC1a.

Supplementary Fig. 6: Cryo-EM processing workflow of EGTA data set.

Supplementary Fig. 7: EGTA, closed, hBASIC cryo-EM map analysis.

Supplementary Figure 8. EGTA, expanded, hBASIC cryo-EM map analysis.

Supplementary Figure 9. Example traces and statistical analysis of IC_{50}.

Supplementary Figure 10. Current-voltage and conductance-voltage analysis.

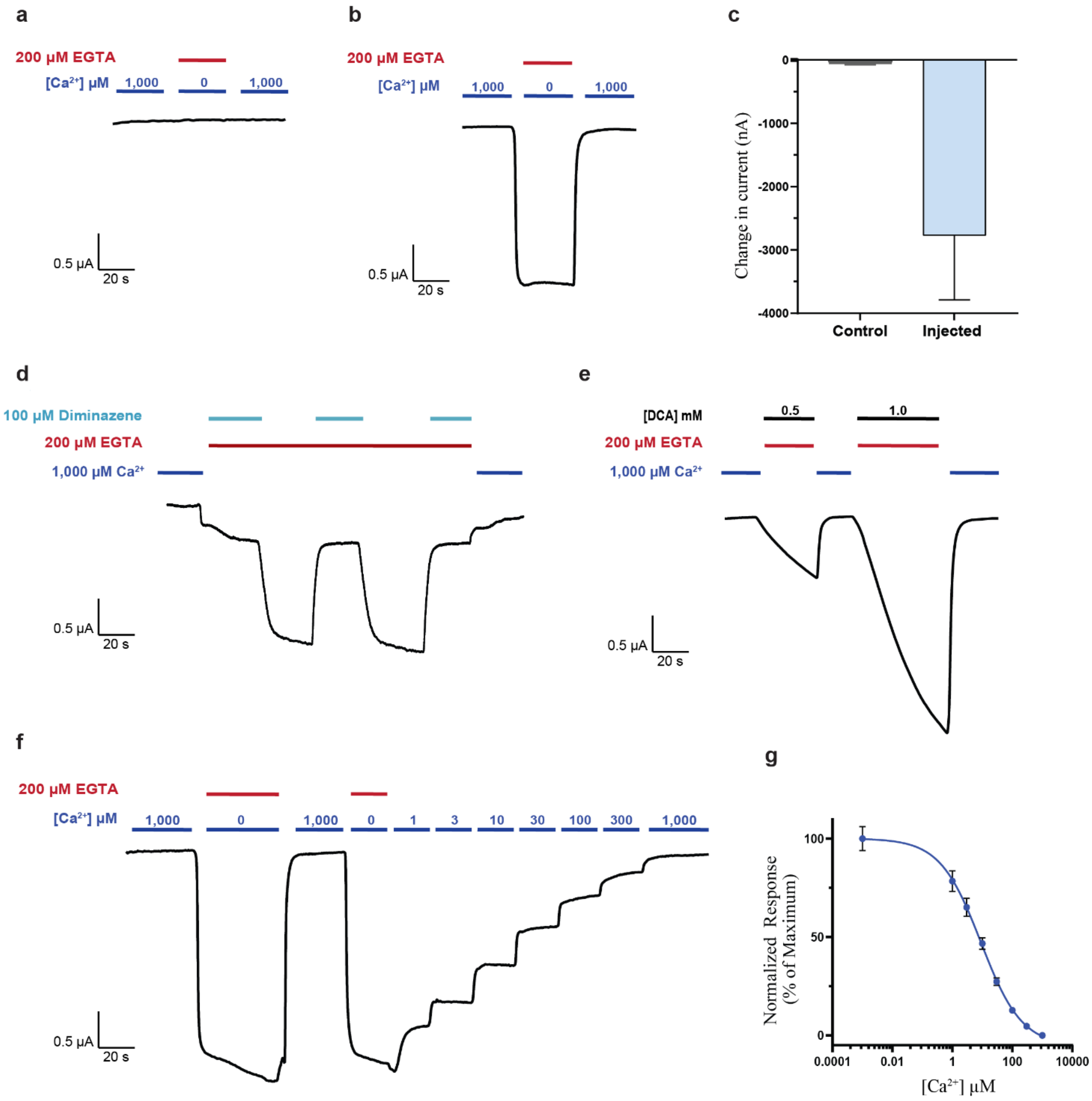

Supplementary Fig. 1. Function of hBASIC construct. (a-b) Example recording of **(a)** oocyte control and **(b)** oocyte expressing hBASIC when recorded in the presence of 1 mM Ca^{2+} or 0.2 mM EGTA. **(c)** Change in current (nA) upon Ca^{2+} removal from the bath between control oocytes and hBASIC injected oocytes. **(d)** hBASIC's inhibitor, diminazene, attenuates Ca^{2+} chelation activated currents in oocytes expressing hBASIC. **(e)** Ca^{2+} chelation activated currents can be potentiated by increasing amounts of the bile acid, deoxycholic acid (DCA). **(f-g)** There is no significant difference in potency of Ca^{2+} inhibition of

hBASIC by Ca^{2+} when perfusion of [Ca^{2+}] is performed in a 'reverse order' (i.e. increasing [Ca^{2+}], instead of decreasing [Ca^{2+}]). **(f)** A representative trace of the control experiment with the corresponding statistics analysis **(g)** that show the IC_{50} is 8μM ± 2.

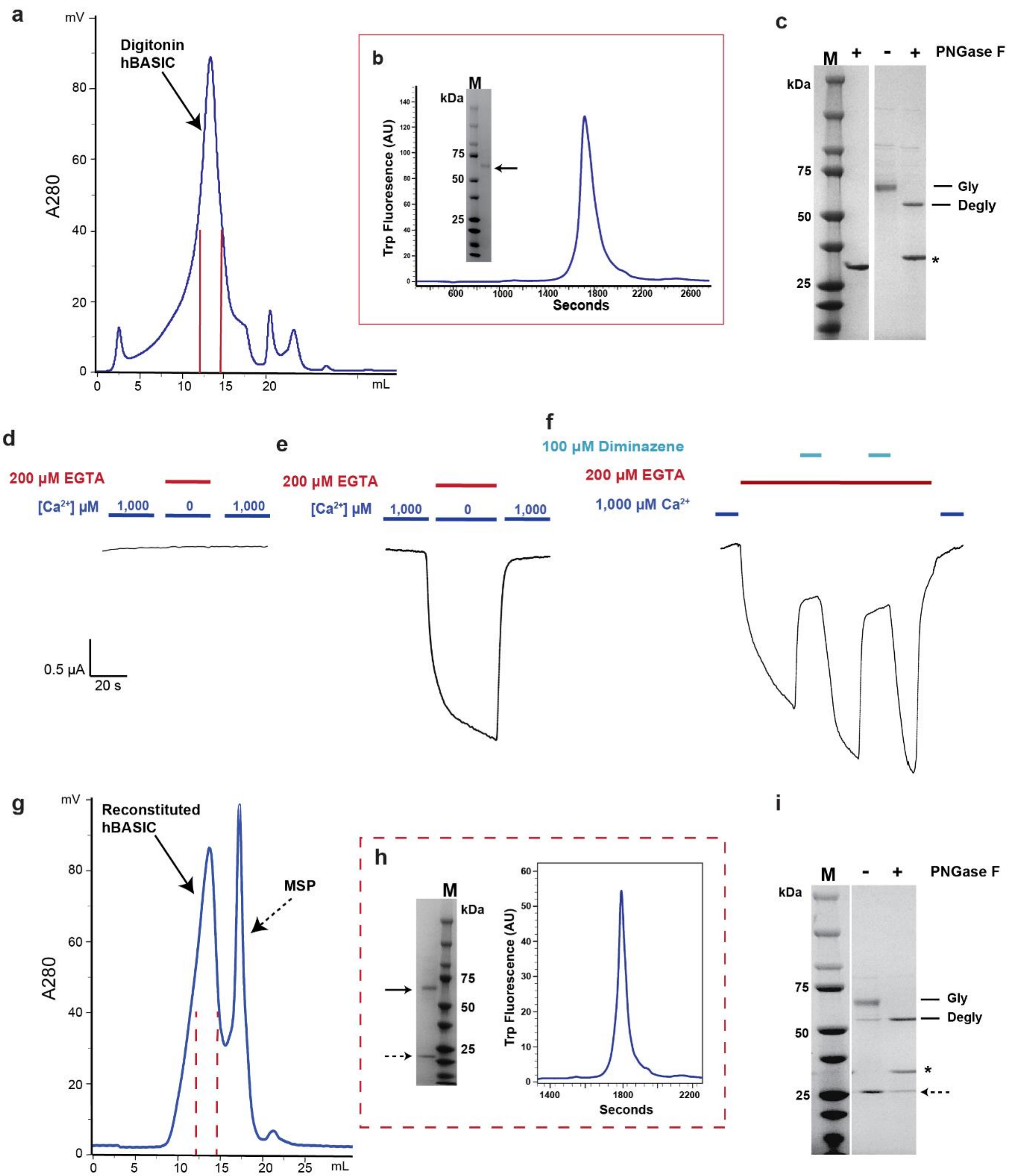

Supplementary Fig. 2. Biochemical and functional characterization of recombinant hBASIC. (a) Size exclusion chromatography (SEC) chromatogram of digitonin extracted hBASIC expressed in HEK293 cells. Solid red lines indicate peak fraction corresponding to hBASIC. **(b)** Fluorescent size exclusion chromatography (FSEC) and SDS-PAGE gel analysis of digitonin hBASIC sample from peak fraction of

SEC elution. **(c)** SDS-PAGE analysis of digitonin solubilized hBASIC with and without PNGase F treatment. Lines indicate glycosylated and deglycosylated hBASIC. Star indicates PNGase F. **(d-f)** Representative TEVC current trace recordings of **(d)** uninjected oocytes and **(e-f)** oocytes injected with digitonin purified hBASIC reconstituted into liposomes. Injected oocytes demonstrate inward sodium currents in response to the removal of Ca^{2+} via EGTA chelation. **(f)** The EGTA chelated currents are attenuated with hBASIC pore blocker, diminazene. **(g)** Example SEC chromatogram of nanodisc reconstituted hBASIC. Solid arrow represents nanodisc embedded hBASIC, while a dashed arrow represents membrane scaffold protein (MSP). Elution fraction indicated with dashed red lines. **(h)** SDS-page and FSEC analysis of nanodisc embedded hBASIC from SEC elution. **(i)** SDS-PAGE analysis of nanodisc-embedded hBASIC with and without PNGase F treatment. Lines indicate glycosylated and deglycosylated hBASIC. Star indicates PNGase F. Dashed arrow indicates MSP protein.

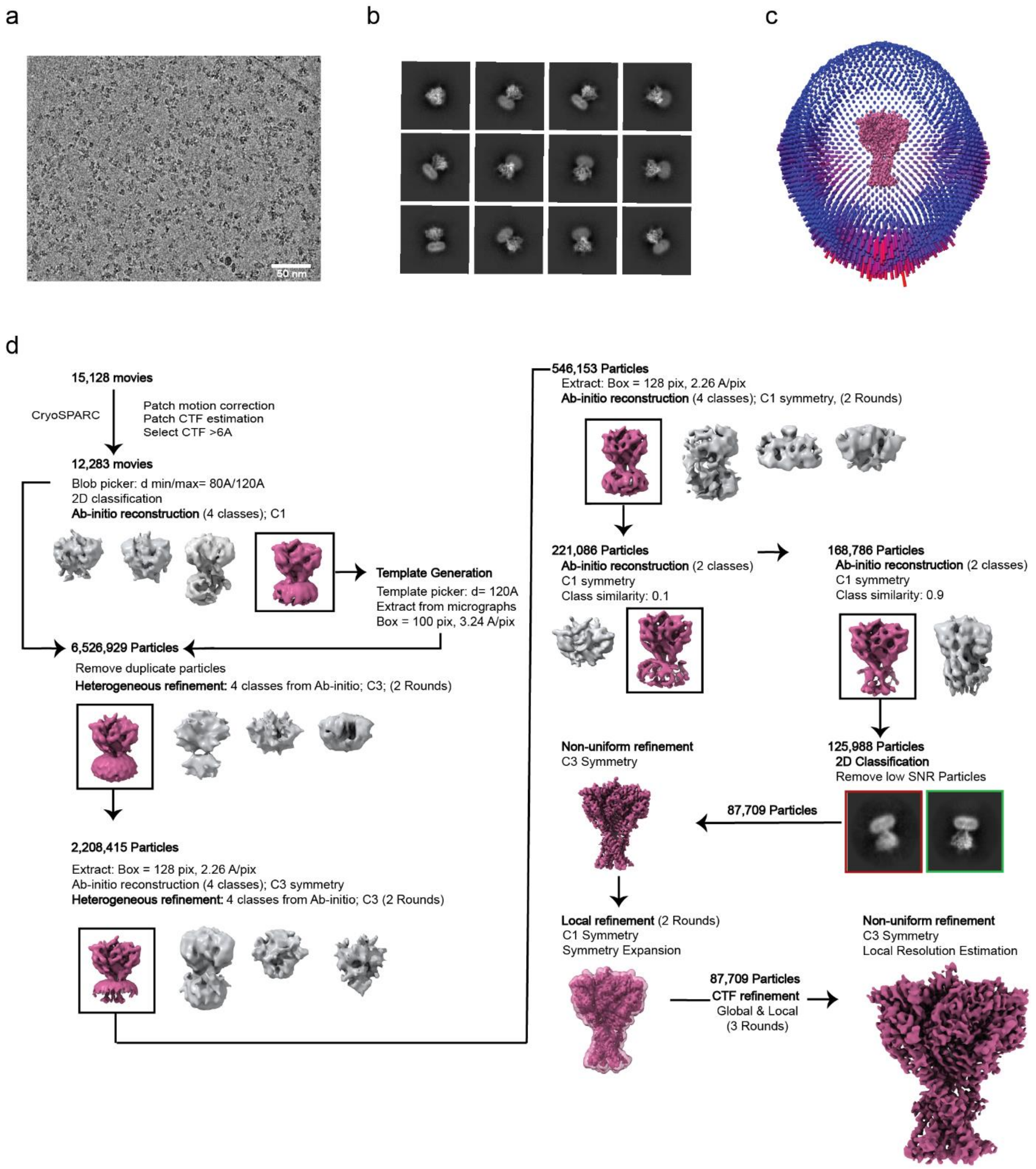

Supplementary Fig. 3. Cryo-EM processing pipeline for 2 mM Ca^{2+} data set. (a) Representative micrograph from data set. **(b)** Selected 2D classes and **(c)** angular distribution of particles included in the final map. **(d)** Data processing workflow.

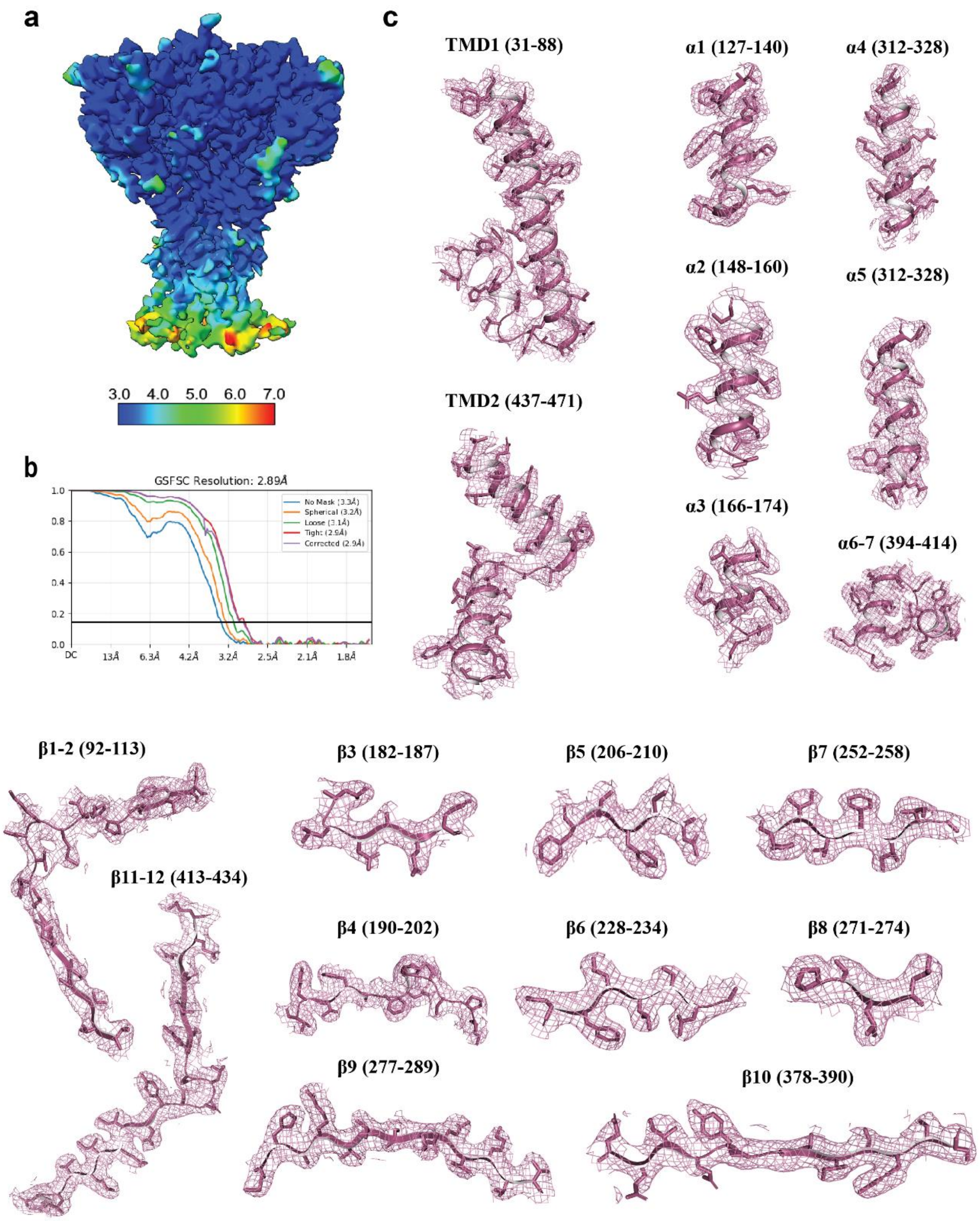

Supplementary Fig. 4. 2mM Ca^{2+} hBASIC cryo-EM map analysis. (a) Local resolution and **(b)** Fourier shell correlation (FSC) plot of 2mM Ca^{2+} hBASIC map. **(c)** Density associated with TMDs, α-helices, and β-sheets. Isomesh map features are contoured at 6.0 σ and within 2Å associated with each feature.

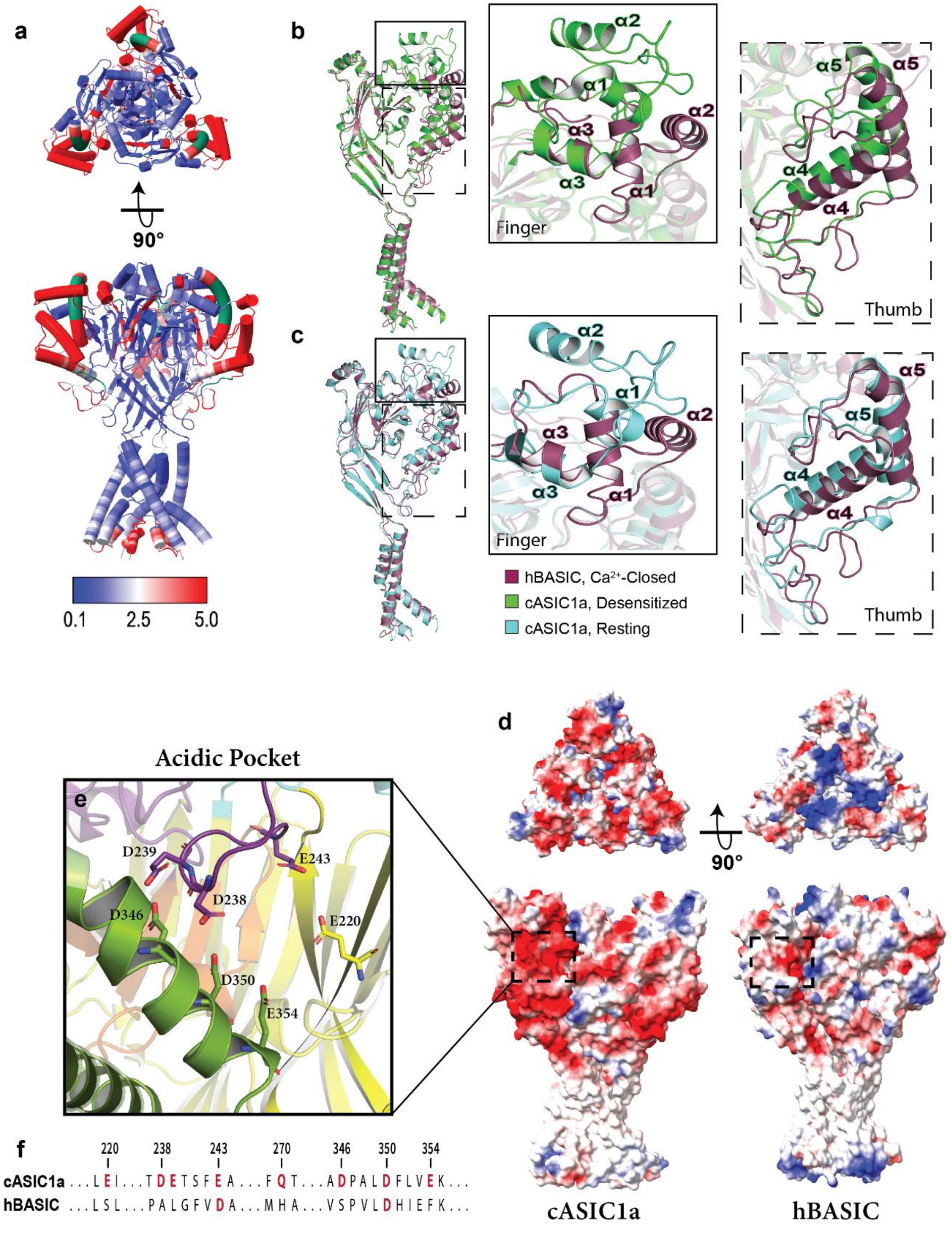

Supplementary Fig. 5. Comparison of hBASIC to cASIC1a. (a) rmsd of Ca^{2+} hBASIC in comparison to cASIC1a, desensitized [PDB:6VTK]. Green represents unique inserts, Blue = high conservation, Red = low conservation. **(b-c)** hBASIC with Ca^{2+} map superimposed onto cASIC1a desensitized [PDB:6VTK] **(b)** and

resting [PDB: 6VTL] **(c)**, highlighting regions of low conservation, the finger and thumb. **(d)** Models of cASIC1a and hBASIC colored by electrostatic potential, with red representing regions of negative potential through blue for positive potential. A dashed box indicates the acidic pocket of cASIC1a and the equivalent region in hBASIC. **(e)** The acidic pocket of cASIC1a, a pocket formed by intersecting regions of the finger, thumb, and palm domain. **(f)** Sequence comparison of the acidic pocket between cASIC1a and hBASIC, showing lack of sequence conservation of these acidic residues.

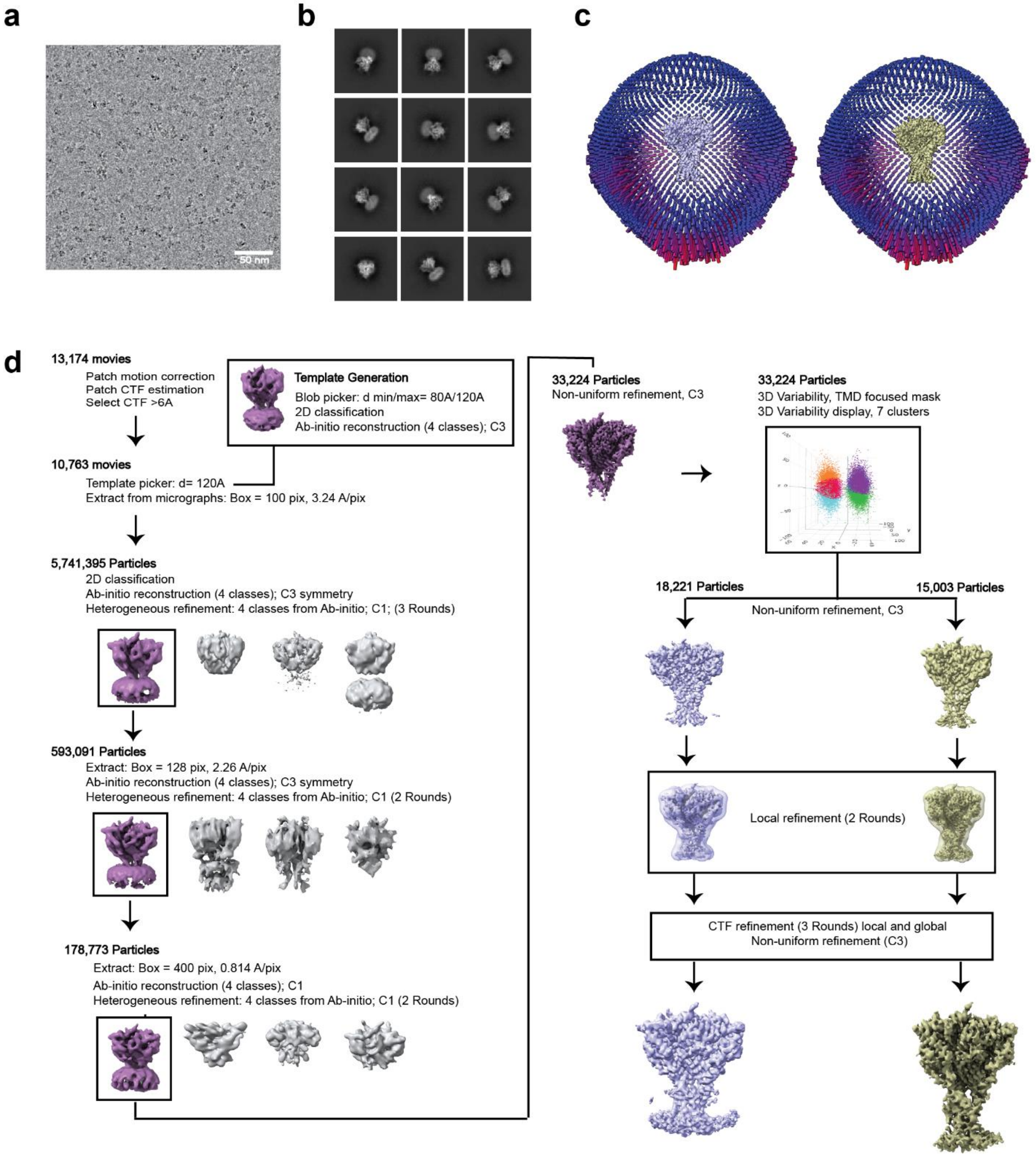

Supplementary Fig. 6. Cryo-EM processing workflow of EGTA data set. (a) Representative micrograph from data set. **(b)** Selected 2D classes and **(c)** angular distribution of particles included in the final maps. **(d)** Data processing workflow.

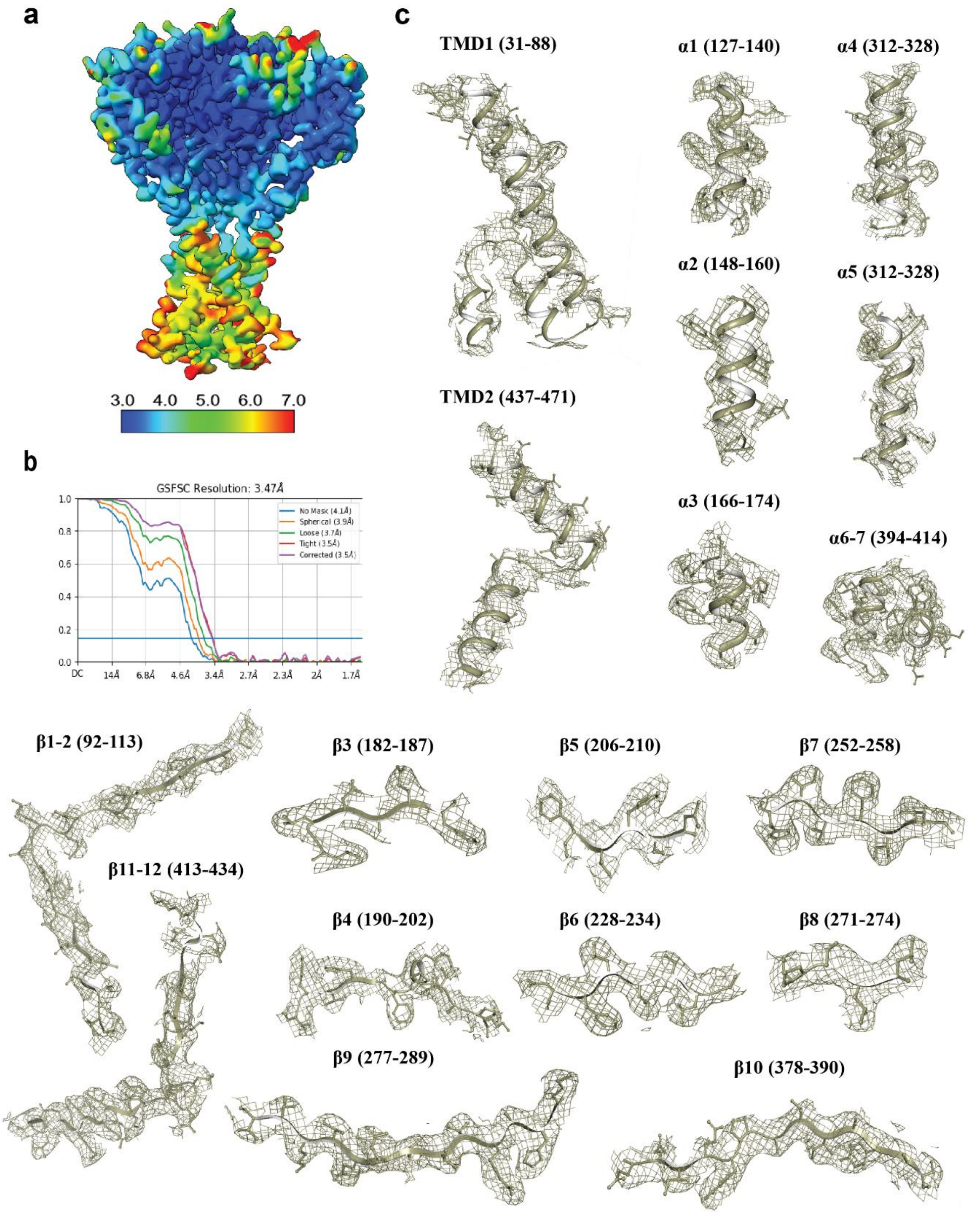

Supplementary Fig. 7. EGTA, closed, hBASIC cryo-EM map analysis. (a) Local resolution and **(b)** Fourier shell correlation (FSC) plot of EGTA, closed, hBASIC map. **(c)** Density associated with TMDs, α-helices, and β-sheets. Isomesh map features are contoured at 4.0 σ and within 2Å associated with each feature.

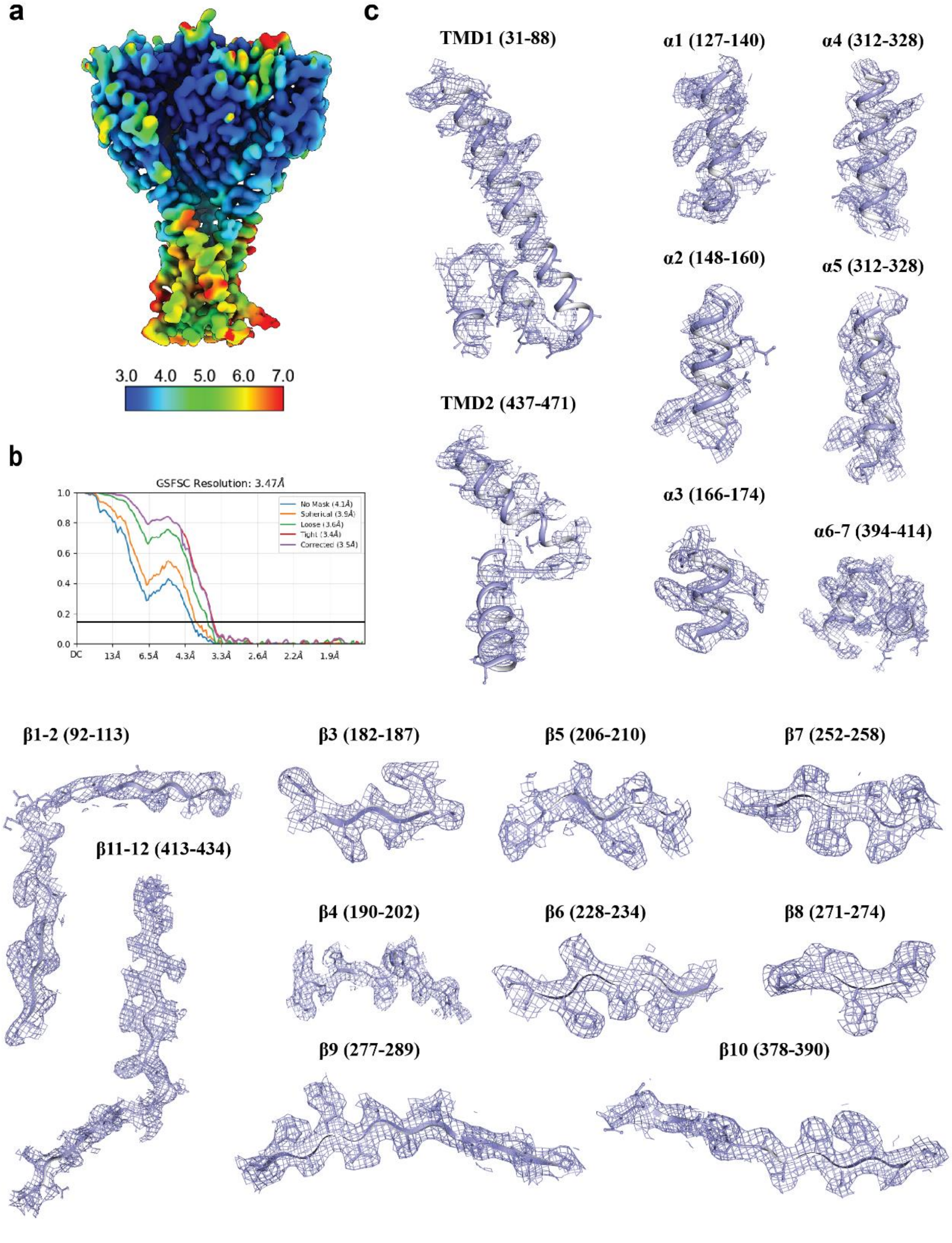

Supplementary Fig. 8. EGTA, expanded, hBASIC cryo-EM map analysis. (a) Local resolution estimation of EGTA, expanded map. **(b)** Fourier shell correlation (FSC) plot of corresponding map. **(c)** Density, contoured at 4.0 σ and within 2Å associated with TMDs, α-helices, and β-sheets.

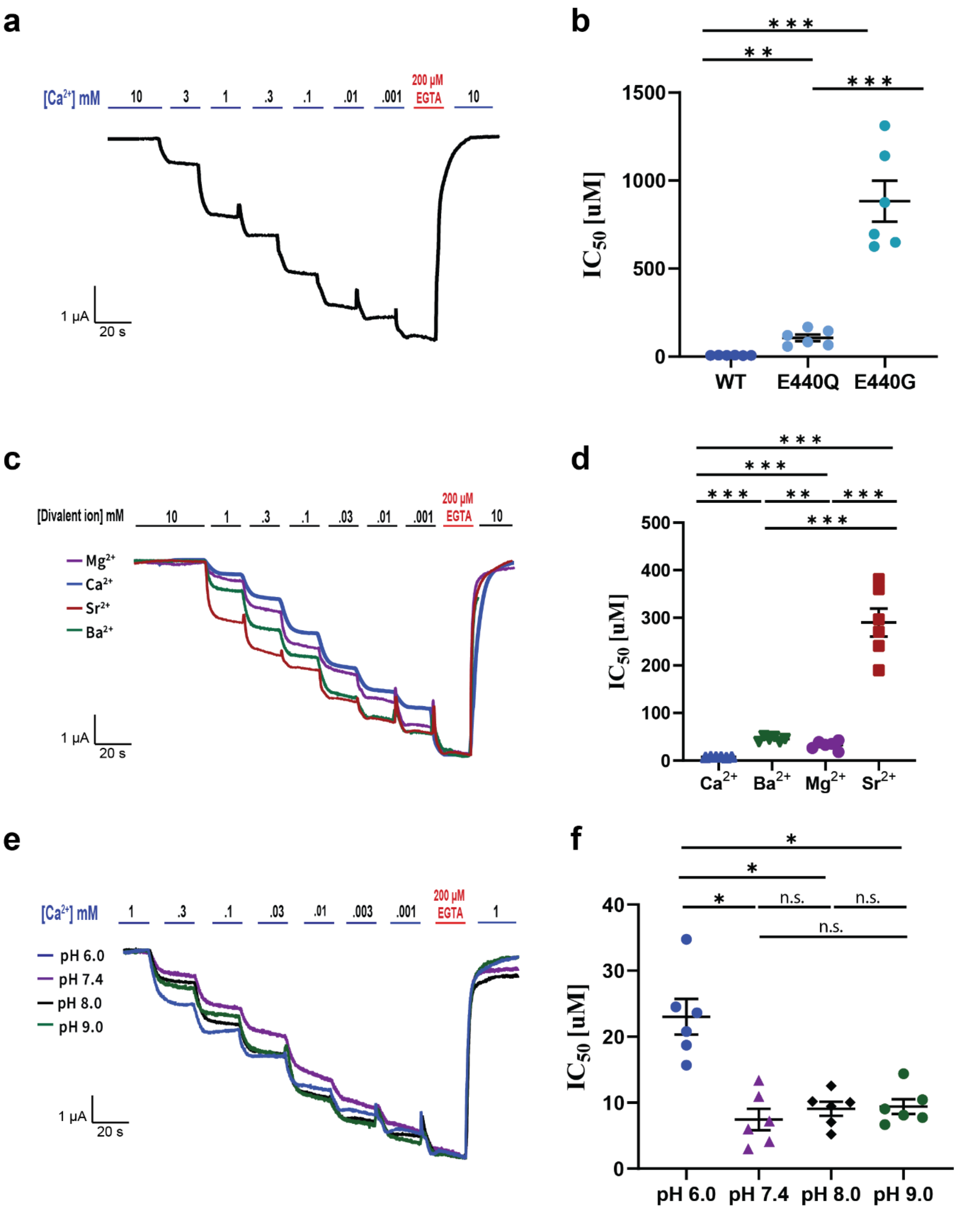

Supplementary Fig. 9. Example traces and statistical analysis of IC_{50}. (a) Example recording of an inhibition dose-response curve of Ca^{2+} of an oocyte injected with mutant E440Q. **(b)** Statistical analysis of

IC_{50} of WT, and mutants, E440Q and E440G. WT versus E440Q, P= 1.4 E-4; WT versus E440G, P=9.9 E-6, E440Q versus E440G, P=3.1E-5. **(c)** Example traces of dose-response recordings of oocytes expressing hBASIC for inhibition by Mg^{2+}, Ca^{2+}, Sr^{2+}, and Br^{2+}. **(d)** Statistical analysis of IC_{50} of inhibition by divalent ions. Ca^{2+} versus Ba^{2+}, P= 1.1 E-7; Ca^{2+} versus Mg^{2+}, P=2.7 E-5, Ca^{2+} versus Sr^{2+}, P=1.2 E-6, Ba^{2+} versus Mg^{2+}, P=4.7 E-4, Ba^{2+} versus Sr^{2+}, P=7.3 E-6, Mg^{2+} versus Sr^{2+}, P=2.9 E-6. **(e)** Example traces of dose-response recordings of oocytes expressing hBASIC for inhibition by Ca^{2+} at pH 6.0. **(f)** Statistical analysis of IC_{50} of inhibition by Ca^{2+} at different pH values. pH6 versus pH7.4, P= 0.019; pH6 versus pH8, P=0.026, pH6 versus pH9, P=0.028, pH7.4 versus pH8, P=0.127, pH7.4 versus pH9, P=0.103, pH8 versus pH9, P=0.42. **(b, d, f)** Statistical significances are shown as $*P < 0.05$, $**P < 0.01$, $***P < 0.001$. Significances were evaluated using two-tailed Student's *t*-tests.

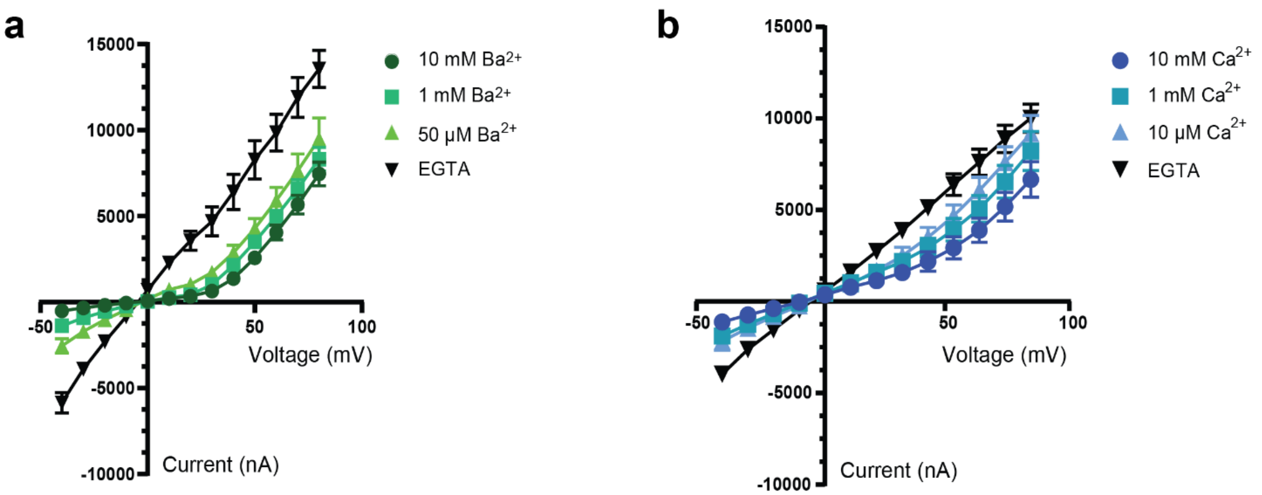

Supplementary Fig. 10. Current-voltage and conductance-voltage analysis. (a) Current-voltage (IV) plot of oocytes (n=7) injected with wild-type hBASIC, recording various amounts of Br^{2+}. **(b)** IV plot of oocytes (n=7) injected with mutant hBASIC, E440Q, recording various amounts of Ca^{2+}.
